# Psychometric properties of the Thai version of the Illness-Specific Social Support Scale Short Version-8 (ISSS-8) among hematological malignancy patients in the Northeastern region of Thailand: A multicenter study

**DOI:** 10.1371/journal.pone.0341756

**Published:** 2026-07-31

**Authors:** Ueamporn Summart, Monthida Sangruangake, Chaliya Wamaloon, Acharaporn Moungmultri, Muhamad Zulfatul A’la

**Affiliations:** 1 Faculty of Nursing, Roi Et Rajabhat University, Roi Et, Thailand; 2 Faculty of Nursing, Udon Thani Rajabhat University, Udon Thani, Thailand; 3 Ubon Ratchathani Cancer Hospital, Ubon Ratchathani, Thailand; 4 Roi Et Hospital, Roi Et, Thailand; 5 Faculty of Nursing, Universitas Jember, Jember, Indonesia; Chulalongkorn University Faculty of Psychology, THAILAND

## Abstract

Social support, which is an essential aspect influencing health, has resulted in the development of several approaches for assessment in cancer patients. The Illness-Specific Social Support Scale Short Version-8 (ISSS-8) effectively evaluates social support among diverse patient demographics; however, its psychometric validity has yet to be established in Asian cultural contexts. The objectives of this study were to translate and culturally adapt the ISSS-8 for the Thai setting and to assess its psychometric properties in patients with hematological malignancies (HMs). This study employed a convenience sampling method to select patients with HMs undergoing hospitalization at three tertiary institutions in Northeastern Thailand. Psychometric testing was conducted following the translation and cross-cultural adaptation of the ISSS-8 into Thai. A total of 350 patients were recruited. Participants were randomly divided into two groups for exploratory factor analysis (EFA) (n = 200) and confirmatory factor analysis (CFA) (n = 150). The EFA of the eight items yielded a loading from a two-factor model comprising Positive Support and Detrimental Interactions, which explained 82.44% of the variance. Cronbach’s alpha (0.80) and item–total correlations (rho = 0.33–0.65) demonstrated acceptable reliability of the ISSS-8, while test–retest reliability was high (ICC = 0.924-–0.934). The average variance extracted (AVE) demonstrated convergent validity for all ISSS-8 subscales, with AVEs ranging from 0.76 to 0.80. Moreover, the total ISSS-8 scale demonstrated a statistically significant but weak positive correlation with the Stanford Inventory of Cancer Patient Adjustment (r = 0.244, p < 0.001), while the Detrimental Interactions subscale was significantly and weak negatively correlated with it (r = 0.237, p < 0.001). These findings suggest that the ISSS-8 captures distinct psychosocial and illness-specific relational constructs that differ from coping self-efficacy and psychological adjustment measured by the SICPA. The ISSS-8 is a short, accurate, and valid instrument for measuring social support in Thai patients with HMs. As a result, healthcare professionals can use the ISSS-8 to assess social support in both research and clinical settings. The findings emphasize the necessity of integrating social support evaluation into cancer management and family-centered care, thereby supporting comprehensive and adaptive healthcare delivery in Thailand.

## Introduction

Hematological malignancies (HMs), including leukemia and lymphoma, pose a considerable global health challenge, with incidence rates rising since 1990, culminating in approximately 1.34 million cases in 2019 [1]. Chemotherapy is the primary and indispensable treatment for the majority of hematological malignancies due to the systemic nature of the disease and the accelerated growth rate inherent to malignant hematological cells [2]. Treatment for these types of malignancies usually involves long, intense chemotherapy regimens, which have been associated with extensive physical and mental distress, including extreme fatigue, anxiety, and depression, as well as a substantial decline in daily functioning and quality of life (QOL) [3]. Considering the severity and duration of treatment, it is crucial to employ appropriate psychological assessments to facilitate patient adjustment to illness and treatment and optimize clinical outcomes. Some cancer patients are able to cope with their diagnosis and treatment, whereas others experience negative emotional responses, including lethargy, fragility, melancholy, and anxiety, particularly at the initial stage [4]. Incapacitation, frailty, sadness, trauma, panic, and concerns over personal survival frequently follow these emotions [5].

Social support is widely acknowledged as a crucial factor influencing psychological well-being, stress adaptation, and health outcomes among various populations [6]. It has been consistently associated with psychological adjustment, coping, and health-related quality of life among patients with cancer and their family caregivers [7,8]. Growing evidence indicates that adequate social support is associated with reduced emotional distress and improved psychological adjustment, whereas conflict-laden or problematic support may exacerbate stress and negatively influence coping and adaptation [7,9]. In patients with HMs, who frequently undergo intensive therapies including chemotherapy, stem cell transplantation, and prolonged hospitalization, approximately 40–60% of patients experience increased mental distress [10]. Previous research indicates that cancer patients with substantial social support exhibit improved QOL and reduced mortality, whereas those lacking sufficient support experience inferior oncological outcomes, expedited disease progression, and diminished overall survival [11,12]. Consequently, numerous researchers regard social support as a significant indicator of health, which has resulted in the development of various methods to assess social support in cancer patients.

In the Thai setting, the revised Thai version of the Multidimensional Scale of Perceived Social Support (r-Thai MSPSS) has been validated among Thai medical students and has been widely used to assess perceived social support [13]. This tool offers advantages including brevity, established reliability, and comparability across studies [13,14]. However, this instrument’s focus on broad, positively perceived help may limit its sensitivity to the complex, context-specific dimensions of social support experienced by cancer patients [15].

While general social support instruments such as the MSPSS evaluate broad perceptions of family, friend, and significant-other support, they may inadequately capture illness-specific interpersonal experiences commonly encountered in oncology settings. Patients with HMs frequently experience complex support-related interactions associated with their illness and treatment, including overprotectiveness, excessive illness-related worry, unsolicited medical advice, and criticism regarding coping behaviors. General social support instruments often overlook such interactions, which can significantly impact psychological adjustment, coping capacity, and emotional distress. The Illness-Specific Social Support Scale Short Version-8 (ISSS-8) addresses this limitation by separately assessing both Positive Support and Detrimental Interactions, thereby providing a more clinically sensitive evaluation of social support among cancer patients [7,9]. The ISSS-8 has demonstrated satisfactory psychometric performance across diverse patient populations, particularly among individuals with cancer and chronic illnesses [7,16–18]. Previous validation studies consistently support its two-factor structure comprising Positive Support and Detrimental Interactions, with strong internal consistency and clinical relevance in oncology settings [7,17].

However, despite its established utility in predominantly Western populations, the psychometric properties of the ISSS-8 have not yet been examined within Asian cultural contexts, including Thailand. Validation studies of the ISSS-8 also remain limited among patients with HMs. Cross-cultural validation is important because sociocultural factors may influence how illness-related social support is perceived and experienced. In Thai collectivist culture, family involvement in illness care is highly emphasized, which may shape patterns of social support differently from those observed in Western settings where the ISSS-8 was originally developed [19]. Furthermore, psychometric adaptation of psychosocial instruments within Thai populations remains necessary to ensure cultural relevance and measurement validity [20]. Misinterpretation of illness-related support dynamics resulting from the use of an unvalidated instrument may compromise the accuracy of psychosocial assessment and supportive care planning [21]. Moreover, understanding culturally contextualized support systems may help healthcare professionals provide more individualized and family-centered care for patients with HMs and their caregivers [22]. Therefore, the rising incidence and prevalence of hematological malignancies necessitate validation of the psychometric properties of the ISSS-8 to ensure accurate assessment of illness-related social support and appropriate psychosocial care planning. The present study aimed to translate and culturally adapt the ISSS-8 into Thai and evaluate its psychometric properties among patients with hematological malignancies, including content validity, construct validity, convergent validity, and reliability.

## Materials and methods

### Study design and participants

This study employed a multicenter, methodological design using data from a larger project, namely “Self-care behaviors and the relationship between health literacy, self-efficacy, social support, and self-care behaviors among patients with lymphoma receiving chemotherapy.” This study utilized a convenience sampling approach to select patients with HMs receiving treatment or hospitalized at three tertiary hospitals in the Northeastern region of Thailand from August 1 to December 31, 2025, using self-assessment questionnaires. The criteria for inclusion were the following: 1) a diagnosis of leukemia, lymphoma, or multiple myeloma and undergoing chemotherapy; 2) aged ≥ 18 years; 3) adequate reading and writing skills in Thai to complete the survey; and 4) willingness to participate. The criteria for exclusion were the following: 1) simultaneous treatment for other malignancies; 2) being in the terminal stage of the illness; and 3) insufficient cognitive or physical ability to participate in the study.

This study involved the translation and cultural adaptation of the ISSS-8 into Thai to assess social support among patients with HMs in Thailand. The original author authorized the translation and adaptation of the ISSS-8 for use in Thailand via email. The research received approval from the university’s ethics committee and was conducted in compliance with recognized ethical standards.

### Translation and cultural adaptation

The Thai version of the ISSS-8 was translated and culturally adapted from the English version of the ISSS-8, based on the short-version structure reported by Ramm and Hasenbring [17]. The translation and cross-cultural adaptation process followed internationally recognized guidelines for patient-reported outcome measures, particularly the ISPOR Principles of Good Practice for the Translation and Cultural Adaptation Process [23]. First, forward translation from English to Thai was independently conducted by two bilingual translators, including one professional medical translator and one senior oncology nurse specialist familiar with psychosocial oncology terminology. Discrepancies between the translated versions were reconciled through consensus discussion with a third independent researcher. Subsequently, backward translation into English was independently performed by two native English-speaking translators who were blinded to the original questionnaire and study objectives. An expert committee consisting of two oncologists, two clinical psychologists, and one psychometrician reviewed all translated versions to evaluate semantic, conceptual, idiomatic, and cultural equivalence between the original and Thai versions. Cognitive debriefing interviews were then conducted with 30 patients with HMs to assess clarity, comprehensibility, readability, and cultural relevance of the items. Minor linguistic modifications were subsequently made prior to final psychometric testing.

### Study instruments

The first section of the questionnaire was composed of patient demographic data, including gender, age, educational level, marital status, occupation, income, type of HM, stage of cancer, and chemotherapy regimen.

The Thai version of the Illness-Specific Social Support Scale Short Version-8 (ISSS-8) was translated and culturally adapted from the original English version, following internationally recognized guidelines for cross-cultural adaptation of patient-reported outcome measures [23]. The original ISSS-8 was developed by Ramm and Hasenbring [17] based on the Illness-Specific Social Support Scale proposed by Revenson et al. [9]. The eight-item ISSS-8 assesses patients’ perceptions of illness-related positive social support and detrimental social interactions associated with chronic illness. The instrument consists of two subscales: Positive Support (four items) and Detrimental Interactions (four items). Items are rated on a five-point Likert scale ranging from 0 (“never”) to 4 (“always”), with each subscale score ranging from 0 to 16. Higher scores on the Positive Support subscale indicate greater perceived supportive interactions, whereas higher scores on the Detrimental Interactions subscale indicate more frequent negative or unhelpful interactions related to illness [16]. The original version demonstrated acceptable internal consistency, with Cronbach’s alpha coefficients of 0.88 for Positive Support and 0.68 for Detrimental Interactions [17].

The Thai version of the Stanford Inventory of Cancer Patient Adjustment (SICPA) was utilized to measure coping self-efficacy and psychological adjustment [24]. This 38-item self-report questionnaire assesses patients’ confidence in managing cancer-related challenges across multiple dimensions, including emotional management, communication with medical staff, and functional activities. Each item is rated on a 10-point Likert scale ranging from 1 (not at all confident) to 10 (completely confident), with total scores ranging from 38 to 380. Higher scores indicate greater coping self-efficacy and adjustment. A sample item includes the following: “How confident are you that you can manage the physical side effects of your chemotherapy?” In the current study, the internal consistency of the SICPA was satisfactory (Cronbach’s alpha = 0.87).

### Sample size calculation

The sample size for confirmatory factor analysis (CFA) and structural equation modeling (SEM) was determined using the N: q rule, representing the ratio of participants (N) to model parameters (q), in accordance with Kline’s recommendation of a 10:1 ratio [25]. Considering the number of items in the ISSS-8 (n = 8), a minimum required sample size of 80 participants was established. Consequently, 350 patients with HMs were included in this cross-sectional study. To mitigate model overfitting, exploratory factor analysis (EFA) and confirmatory factor analysis (CFA) were conducted on participants randomly allocated into two groups (group 1, n = 200; group 2, n = 150).

### Statistical analysis

All statistical analyses in this study were performed utilizing IBM SPSS and AMOS version 26. The demographic data of the participants were characterized using descriptive statistics, encompassing means and standard deviations for continuous variables, as well as frequencies and percentages for categorical data. Data were checked for missing values before analysis, and only fully answered questionnaires were included in the analysis. In addition, the skewness and kurtosis of all ISSS-8 items were initially evaluated to determine normality.

The evaluation of floor and ceiling effects was conducted by analyzing the proportion of respondents who achieved scores at the floor (minimum score) and ceiling (maximum score), respectively. We established that a floor or ceiling effect would be considered relevant based on the empirical threshold of 15% and a cumulative ceiling or flooring of 50%, as proposed. Floor and ceiling effects can indicate that extreme items are missing at either end of the scale, which can possibly limit its validity [26]. Additionally, the discriminative capacity of the items was evaluated by corrected item–rest polyserial correlation, with acceptable indices exceeding 0.20 [27].

Parallel analysis (based on principal component analysis) was employed with sample group 1 (n = 200) to ascertain the number of components in the exploratory factor analysis (EFA) of the ISSS-8 measurement model. To accommodate the expected conceptual relationship between the social support dimensions, the component structure was subsequently examined through principal component analysis with an oblique (Promax) rotation. Factor loadings below 0.50 were considered negligible, and item cross-loadings exceeding 0.20 were sequentially removed [28]. Additionally, the rescaled component loadings from the pattern matrix were employed to compute the average variance extracted (AVE) and composite reliability (CR). After establishing the principal component analysis results, construct validity was evaluated using CFA with sample group 2 (n = 150). The first-order two-factorial model of the ISSS-8 and the second-order unique-factor model of the ISSS-8 were evaluated. A robust maximum likelihood estimator was utilized to address multivariate non-normality. Hu and Bentler’s criteria [29] for various fit indices were employed to assess the fit of the proposed model to the data. The chi-square test statistic was employed; however, due to its sensitivity to sample size, additional fit indices were assessed: (a) the comparative fit index (CFI ≥ 0.90 signifies a good fit); (b) the root mean square error of approximation (RMSEA ≤ 0.08 denotes an acceptable fit); and (c) the standardized root mean square residual (SRMR ≤ 0.08 indicates an adequate fit). A chi-square difference test, utilizing the Satorra-Bentler scaled chi-square test [30], was employed to compare the first-order two factorial model of the ISSS-8 with the second-order unique-factor model of social support. Along with the CFA, the Kaiser-Meyer-Olkin (KMO) measure of sampling adequacy and Bartlett’s test of sphericity were conducted to demonstrate factorability [28].

To maximize statistical stability and representativeness, reliability analyses were performed using the total sample (N = 350). Reliability was evaluated using both CR and Cronbach’s alpha. CR values surpassing 0.60 and Cronbach’s alpha values exceeding 0.70 were considered satisfactory for all subscales. Additionally, item-total correlations were examined using a threshold of 0.30 or greater to ensure each item contributed meaningfully to its respective scale without redundancy [31].

The convergent validity of the ISSS-8 was evaluated based on AVE, which indicated the proportion of variance explained by each latent variable derived from all items within that variable. An AVE value exceeding 0.50 signifies that the latent variable exhibits adequate convergent validity [29]. Likewise, the square root of AVE for each sub-dimension should be greater than the correlation coefficients between sub-dimensions to verify discriminant validity [29].

Concurrent validity was measured using Pearson’s correlation coefficient. We hypothesized a positive correlation between the ISSS-8 and self-efficacy as measured by the SICPA, assuming that high correlation implies excellent concurrent validity and suggests that the two scales assess similar concepts [32]. Following Cohen’s criteria, the correlation between the variables was classified as small, medium, or large, with correlation coefficients greater than 0.10, 0.30, and 0.50, respectively [33].

Discriminant validity was assessed by comparing the square root of AVE with the correlation coefficient of observed variables. A square root of AVE greater than the correlation coefficient signifies stronger discriminant validity [34]. In addition, to evaluate the discriminant validity of the Thai Illness-Specific Social Support Scale (Thai ISSS-8), two rigorous approaches were utilized: a cross-loading analysis and the Heterotrait-Monotrait (HTMT) ratio of correlations. An HTMT ratio below 0.85 was adopted as the threshold to indicate strict discriminant validity [35].

Test–retest reliability was assessed in a sub-sample of 30 participants to determine the intraclass correlation (ICC). Reliability was considered adequate when ICC values surpassed the threshold of 0.70 [36].

### Ethical considerations

The study followed the ethical standards and principles outlined in the Declaration of Helsinki. Ethical clearance for the study protocol was granted by the Roi Et Rajabhat University Ethics Committee for Human Research (Reference No. RERU-EC 058/2568), Roi Et Hospital (Reference No. RE 130/2568), Udon Thani Hospital (Reference No. UDH-REC 50/2568), and Ubon Ratchathani Cancer Hospital (Reference No. EC 005/2025). Each study participant provided informed written consent to participate in this investigation. Furthermore, each participant was assured of the confidentiality of his or her personal information.

## Results

### General information of the participants

Participant characteristics are detailed in Table 1. Approximately 73.1% of the participants were male (n = 256), while 26.9% were female (n = 94), with ages ranging from 19 to 91 years (M = 53.32; SD = 12.30). Over 47.7% of the participants had attained secondary education. Lymphoma was the predominant diagnosis, accounting for 72.6% of cases, with stage III comprising 35.4%.

**Table 1:** Demographic characteristics of patients with HMs (n = 350).

Baseline characteristic	N	%
Gender		
Male	256	73.1
Female	94	26.9
Age (years)		
Mean (SD)	53.32 (12.30)	
Median (min: max)	54.0 (19:91)	
Education level		
Primary	114	32.6
Secondary	167	47.7
Bachelor’s or higher	69	19.7
Religion		
Buddhism	296	84.6
Christianity	27	7.7
Islam	27	7.7
Occupation		
Agriculture	141	40.2
Civil servant	14	5.2
Employee	118	32.6
Personal business	35	10.0
Others	42	12.0
Household income (baht)		
Less than 10,000	198	56.5
10,000-14,999	92	26.3
15,000-19,999	23	6.8
≥ 20,000	37	10.4
Marital status		
Single	117	33.4
Married	230	65.7
Divorced/separated	3	0.9
Cancer diagnosis		
Lymphoma	254	72.6
Leukemia	96	27.4
Stage of cancer		
I	70	20.0
II	116	33.1
III	124	35.4
IV	30	8.6
Unknown	10	2.9

### Descriptive analysis and item analysis of the ISSS-8

The descriptive analysis of the ISSS-8 items is presented in Table 2. The average scores of the responses to the eight items from all participants ranged from 2.61 to 3.17. For the item analysis, the results showed that all items demonstrated acceptable normality, with skewness and kurtosis values less than ±1. Floor effects were observed for all Detrimental Interactions items, whereas ceiling effects were observed for all Positive Support items. In terms of discriminative capacity, all items had polyserial item-rest correlation coefficients greater than 0.20 (Table 2).

**Table 2:** Distribution of responses and discrimination of the items.

Item	Response (%)					Mean	SD	Item-rest correlation
	0	1	2	3	4			
1	0.0	4.9	11.2	49.2	34.6	3.17	0.06	0.40
2	4.4	0.5	20.0	46.3	28.8	3.07	0.07	0.48
3	0.0	4.9	11.2	49.3	34.6	3.17	0.06	0.40
4	3.9	3.9	16.6	46.8	26.3	3.13	0.06	0.57
5	31.7	12.2	38.5	14.6	2.9	3.39	0.08	0.52
6	32.7	11.7	32.7	15.1	7.8	2.61	0.01	0.69
7	38.0	12.7	33.2	13.7	2.4	2.69	0.96	0.70
8	42.5	17.1	24.9	8.3	7.3	2.70	0.10	0.71

### Exploratory factor analysis

After randomly assigning individuals to two groups (n = 200 for EFA and n = 150 for CFA), parallel analysis was used to determine the appropriate number of components to retain. The initial analysis of the group 1 sample identified two components with eigenvalues greater than one. The two-component solution, utilizing a principal component analysis with an oblique (Promax) rotation, accounted for 82.44% of the total variance. The KMO measure was 0.710, and Bartlett’s test of sphericity was significant (χ^2^ = 1667.21; p < 0.001), indicating that the data was suitable for factor analysis (Table 3).

**Table 3:** Factor loading values for each item of the ISSS-8 and scale-level statistics (n = 200).

ISSS-8 item	Component 1	Component 2
Amongst the people you feel close to, is there someone who...	**Positive Support**	**Detrimental Interactions**
1. Is there for you when you need him/her.	0.949	
2. Gives you comfort	0.875	
3. Talks about important decisions with you	0.949	
4. Spends part of his/her time working some things out for you	0.840	
5. Worries too much about your illness		0.835
6. Gives you information or makes suggestions that you find unhelpful or upsetting		0.925
7. Makes you feel you cannot care for yourself		0.940
8. Tries to change the way you’re coping with your illness in a way you don’t like		0.899
**Scale-level statistics**		
KMO	0.710	
Cronbach’s alpha	0.89	0.91
Eigenvalue	3.45	3.14
% of variance	43.18	39.26
Cumulative %	43.18	82.44

### Confirmatory factor analysis

**Fig 1:**
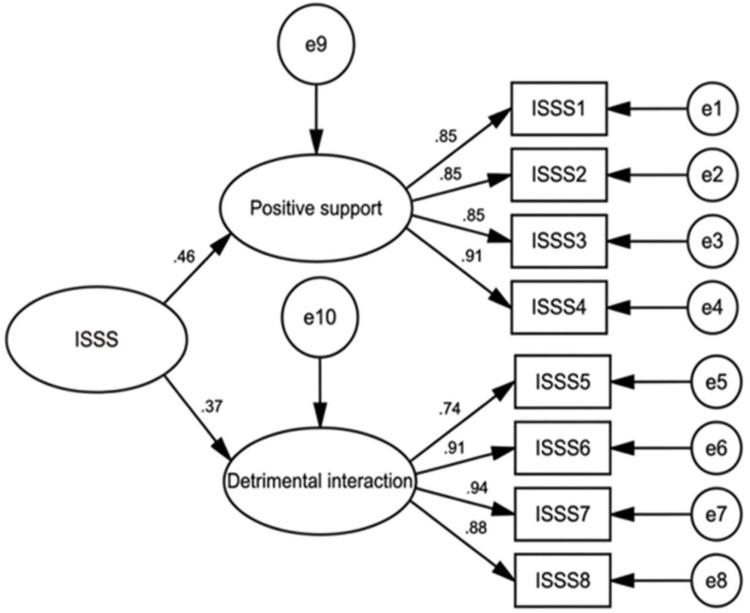
Structural model of the ISSS-8 showing standardized path coefficients.

To assess construct validity, a robust maximum likelihood CFA was conducted to fit the ISSS-8 eight-item measurement model. All eight ISSS-8 items were assigned to two factors consistent with the original structure of the ISSS-8: Positive Support (4 items) and Detrimental Interactions (4 items). A parallel examination of the factor components of the ISSS-8 was performed. The model demonstrated a reasonable fit to the data, as indicated by the five preset fit criteria (χ^2^/df = 1.585; p = 0.550, CFI = 0.99, AGFI = 0.97, TLI = 0.99, and RMSEA = 0.048 (95% CI = 0.023-0.051). All items in the model were significantly loaded onto their respective factors (all p-values < 0.05), except for the factor-constraint item, for which no significance test could be conducted. In addition, all factor loadings were greater than 0.50, indicating that individual ISSS-8 items were suitable for measuring the underlying construct of social support, albeit to varying degrees (Fig 1).

### Reliability analysis

The CR for the two domains of the ISSS-8 ranged from 0.93 to 0.94, demonstrating acceptable reliability. The ISSS-8 also exhibited adequate internal consistency reliability, with Cronbach’s alpha values of 0.80 for the overall scale, 0.89 for Positive Support, and 0.91 for Detrimental Interactions. Strong internal consistency was supported by item-rest correlations for all items, ranging from 0.33 to 0.65, exceeding the recommended threshold of 0.30 (Table 4).

**Table 4:** Reliability analysis of the ISSS-8.

Subscale	Number of items	CR	Cronbach’s alpha	Item-rest correlation	AVE	ICC	HTMT Ratio
Positive Support	4	0.93	0.89	0.33–0.45	0.76	0.924	–
Detrimental Interactions	4	0.94	0.91	0.56–0.65	0.80	0.933	0.199
Overall		–	0.80	–	–	–	–

### Convergent and discriminant validity

Table 4 presents the reliability analysis of the ISSS-8. AVE calculations revealed that all the ISSS-8 subscales exhibited convergent validity (Positive Support AVE = 0.76; Detrimental Interactions AVE = 0.80). The discriminant validity reflects the distinctiveness of the two subscales. The square root of AVE for Positive Support was 0.87, and for Detrimental Interactions it was 0.89, both exceeding the correlation coefficients between the subscales (r = 0.48, 0.37) (Fig 1). These findings confirm the presence of discriminant validity.

Discriminant validity was evaluated using both traditional and contemporary approaches to establish the distinctiveness of the two subscales. According to the Fornell-Larcker criterion, the square root of the AVE for Positive Support (0.87) and Detrimental Interactions (0.89) both exceeded the correlation coefficients between the subscales (r = 0.48 and 0.37, respectively; Fig. 1). To provide more rigorous empirical evidence of distinctiveness, the HTMT ratio of correlations was calculated. The resulting HTMT ratio between Positive Support and Detrimental Interactions was 0.199, falling substantially below the strict conservative threshold of 0.85. Taken together, these findings conclusively confirm that the two domains possess robust discriminant validity and represent conceptually unique dimensions.

### Test-retest reliability

Test-retest reliability refers to the consistency of a measure when administered at different points in time. It is a critical aspect of evaluating the stability and dependability of assessment tools. During a two-week retest assessment, intraclass correlation coefficients (ICCs) were calculated for 30 participants, demonstrating adequate stability. The mean score on the social support scale decreased slightly from 16.16 (SD = 5.48) on Day 1 to 15.80 (SD = 5.13) on Day 14. The ICC value was 0.924 for Positive Support (95% CI = 0.918–0.952, p < 0.001) and 0.933 for Detrimental Interactions (95% CI = 0.913–0.962, p < 0.001).

### Concurrent validity

Concurrent validity of the ISSS-8 and its two subscales was assessed via correlation with the SICPA. The Positive Support subscale demonstrated a small positive correlation with the total SICPA score; however, the association was not statistically significant (r = 0.113). In contrast, the Detrimental Interactions subscale demonstrated a statistically significant weak negative correlation with the total SICPA score (r = −0.237, p < 0.001), indicating that higher levels of negative illness-related social interactions were associated with poorer psychological adjustment. The total ISSS-8 score demonstrated a statistically significant weak positive correlation with the SICPA total score (r = 0.244, p < 0.001 (Table 5).

**Table 5:** Correlation between the ISSS-8 and concurrent measures.

	1	2	3	4
1. Positive Support	1			
2.Detrimental Interactions	0.144**	1		
3. Total ISSS-8	0.672*	0.836*	1	0.244*
4. Total SICPA	0.113**	−0.237*	0.244*	1

* p < 0.001 ** Correlation is not statistically significant.

## Discussion

To the best of our knowledge, this is the first study to translate and validate the psychometric properties of the ISSS-8 for Thai patients with HMS in Northeastern Thailand. Following established international guidelines for the cross-cultural adaptation of patient-reported outcome measures, the ISSS-8 underwent a structured forward-backward translation and cultural adaptation process. The Thai version of the ISSS 8 exhibited acceptable psychometric properties, demonstrating satisfactory structural, convergent, and discriminant validity, as well as strong internal reliability and test-retest reliability. Consequently, these results support the use of the Thai ISSS-8 to assess illness-specific social support within this patient population. [23], The examination of structural validity through EFA and CFA provided evidence supporting the two-factor structure of the ISSS-8 in this population, comprising Positive Support (four items) and Detrimental Interactions (four items). This structure is consistent with the factor structure reported in the German version of the ISSS-8 developed by Ramm and Hasenbring [17]. While the Positive Support subscale reflects beneficial and supportive interactions, such as emotional comfort and practical assistance, the Detrimental Interactions subscale captures unhelpful or distressing interpersonal experiences, including overprotectiveness and criticism. Construct validity of the ISSS-8 was further supported by CFA, with all fit indices demonstrating acceptable model fit based on established psychometric criteria [29,30]. Although the chi-square statistic is known to be sensitive to sample size, the overall pattern of fit indices supported the adequacy of the proposed two-factor model [29]. In addition, all items demonstrated substantial standardized factor loadings, supporting their adequacy in representing the intended constructs of illness-related social support. Collectively, these findings support the utility of the ISSS-8 as a psychometrically sound instrument for assessing social support among patients with hematological malignancies. These findings are consistent with previous validation studies conducted among patients with cancer and chronic illnesses in predominantly Western populations [7,16–18].

The results of this study showed ceiling effects on all items of the Positive Support Subscale and floor effects on all items of the Detrimental Interactions subscale. The ceiling effect in the Positive Support Subscale indicates that the distribution of responses is heavily skewed towards the maximum possible score [26]. The ISSS-8 was designed to assess social support, a factor prevalent in the majority of the population, including those with HMs. The observed ceiling effects in the Positive Support subscale may indicate limited sensitivity for detecting improvements in perceived support among participants already reporting high baseline support levels. Likewise, floor effects existed on the Detrimental Interactions Subscale. This finding may be due to the fact that most participants do not experience high levels of explicitly critical or detrimental interactions related to their illness. Therefore, a large percentage of respondents score at or near the minimum possible score [26]. In addition, the items of each scale exhibited strong discrimination indices. All item-total correlations exhibited a strong positive association with the total score, indicating the scale’s homogeneity. These indices suggest that the Thai-version items of the ISSS-8 could effectively differentiate between high and low scores on this scale. This result is consistent with a study exploring illness-specific positive social support and detrimental interactions in melanoma survivors [7].

The reliability of the ISSS-8 was verified using internal consistency and a test–retest technique. The CR of the two domains of the ISSS-8 varied from 0.93 to 0.94, signifying excellent reliability. The Cronbach’s alpha values in our study indicated that the ISSS-8 total scores and its subscales exhibited strong internal consistency [36]. The high internal consistency indicates that respondents who select high scores for one item are likely to select high scores for other items as well, and conversely. The findings align with results from various validation studies conducted across diverse populations, including cancer and other chronically ill patients [7,16–18,37]. However, these findings are inconsistent with results from a prior study on samples of cancer patient–relative dyads, which reported that internal consistency was partly lower but still acceptable [38]. Furthermore, the item analysis results indicated that the items of each scale exhibited strong discrimination indices. All item-total correlations exhibited a strong positive association with the total score, indicating the scale’s homogeneity. The indices suggest the potential to differentiate between high and low scores on this scale utilizing the Thai-version items of the ISSS-8. The tool’s strong reliability indicates it is well-designed and able to respond efficiently to inquiries, as demonstrated by participant feedback. This evidence confirms that the ISSS-8 is well-regarded among patients with HMs in Thailand. Related research indicates that this assessment instrument provides a high item discrimination index [7]. Moreover, test-retest reliability was assessed over a two-week period with a sample of 30 participants utilizing ICC. The findings exhibited adequate stability, evidenced by an ICC of 0.924 for the Positive Support domain and 0.933 for Detrimental Interactions, signifying excellent dependability in accordance with established standards [36].

The convergent validity of the ISSS-8 was assessed by calculating the AVE for both subscales. The findings indicated that the AVE for all subscales was above 0.50, exceeding the recommended threshold for convergent validity. This approach aligns with recent psychometric validations of perceived social support measures among people with dementia [39]. Additionally, the ISSS-8 exhibited robust discriminant validity across both traditional and contemporary benchmarks. According to the Fornell-Larcker criterion [34], the square root of the AVE for the ISSS-8 subscales consistently surpassed their correlation with the SICPA, indicating that the ISSS-8 successfully captures distinct relational nuances that the social participation measure does not. To provide more stringent empirical support, the HTMT ratio of correlations was evaluated. The calculated HTMT value fell substantially below the conservative 0.85 threshold, further substantiating the distinctiveness of these constructs. Collectively, these rigorous metrics-complemented by high item–rest correlations-provide compelling evidence of the scale’s internal structural validity and its capacity to measure conceptually unique dimensions of the patient experience.

For concurrent validity, the Detrimental Interactions subscale showed a significant negative correlation with self-efficacy as measured by the SICPA. This finding is theoretically consistent, suggesting that unhelpful, critical, or pessimistic illness-related interactions may negatively influence coping self-efficacy and psychological adjustment among patients with hematological malignancies. In contrast, the Positive Support subscale demonstrated a weak and non-significant correlation with SICPA scores. Similarly, although the total ISSS-8 score showed a statistically significant positive correlation with SICPA, the magnitude of the association was relatively weak. The relatively weak correlations observed between the ISSS-8 and SICPA may reflect conceptual distinctions between illness-specific social support and broader psychosocial adjustment constructs measured by the SICPA. These findings are broadly consistent with previous studies suggesting that detrimental illness-related social interactions are associated with poorer psychological outcomes and increased distress among patients with cancer [7,8].

### Strengths and limitations

In this study, we utilized an adequate sample size to analyze the psychometrics of the Thai version of the ISSS-8, alongside a small number of participants to evaluate the test-retest reliability. However, specific limitations that may have impacted the findings must be recognized. Firstly, the use of convenience sampling within three tertiary hospitals in Northeastern Thailand may limit the generalizability and cultural representativeness of the findings. While the sample size was sufficient for this initial psychometric evaluation, the findings may not fully represent patients from other sociocultural and regional populations in Thailand. To address this limitation, future validation studies should employ stratified sampling across multiple regions of Thailand to confirm the stability and applicability of the scale across broader sociocultural contexts. Secondly, our study used a self-report questionnaire, which has inherent limitations related to susceptibility to response and social desirability biases that may affect data accuracy. Future research should incorporate mixed-method approaches-such as combining structured qualitative interviews with quantitative assessments-or utilize external behavioral criteria and clinician-rated scales to supplement and cross-validate self-report data. Additionally, the concurrent validity findings demonstrated relatively weak correlations between the ISSS-8 and the SICPA. This may reflect conceptual differences between illness-specific social support and broader psychosocial adjustment or coping self-efficacy constructs measured by the SICPA. Future studies should consider incorporating additional theoretically related instruments to further examine convergent and concurrent validity across different psychosocial domains. Finally, the study primarily focused on participants without mental illnesses. Future research should expand the scope to include individuals with pre-existing conditions, such as anxiety and depression.

## Conclusion

This research translated and validated the psychometric properties of the Thai version of the ISSS-8, establishing it as a reliable instrument for assessing social support in patients with HMs. Substantial evidence was obtained to confirm its reliability, convergent validity, and construct validity within this population, as verified through CFA and supporting statistical evaluations. The instrument effectively captures all dimensions of social support within this population, closely resembling the original ISSS-8 while incorporating culturally relevant adaptations. Its demonstrated validity and reliability highlight its utility for healthcare professionals in assessing social support levels in patients with HMs in both research and clinical contexts. These findings emphasize the importance of integrating social support assessments into cancer management and family-centered care, thereby enhancing holistic and responsive healthcare delivery in Thailand.

## Supporting information

S1 File Data. (XLSX)
